# Genome Sequence of a Versatile Aromatic Hydrocarbon-Degrading Bacterium, *Arthrobacter* sp. W1

**DOI:** 10.1128/genomeA.00387-15

**Published:** 2015-04-23

**Authors:** Yi Jiang, Yuanyuan Qu, Ping Xu, Hongzhi Tang

**Affiliations:** a State Key Laboratory of Microbial Metabolism and School of Life Sciences and Biotechnology, Shanghai Jiao Tong University, Shanghai, People’s Republic of China; b Joint International Research Laboratory of Metabolic & Developmental Sciences, Shanghai Jiao Tong University, Shanghai, People’s Republic of China; c Key Laboratory of Industrial Ecology and Environmental Engineering, School of Environmental Science and Technology, Dalian University of Technology, Dalian, People’s Republic of China

## Abstract

*Arthrobacter* sp. W1 is a versatile aromatic-degrading strain which can directly or cometabolically degrade various organic pollutants, such as phenol, naphthalene, carbazole, dibenzofuran, and dibenzothiophene. Here, we present a 3.8-Mb draft genome sequence of strain W1, which may provide comprehensive genetic information for the application in environmental pollution remediation.

## GENOME ANNOUNCEMENT

Coking wastewater generated from coke and steel industries is considered one of the most refractory wastewater sources for encompassing various inorganic and organic pollutants (1, 2). Phenol, naphthalene, and heterocyclic compounds such as carbazole (CA), dibenzofuran (DBF), and dibenzothiophene (DBT) coexist and constitute the major portion of organic pollutants in the world. Bioaugmentation has been proven to be an efficient approach to improve the wastewater treatment process (3). In our previous study, a Gram-positive bacterial strain designated W1, which utilizes phenol as the sole carbon source, was isolated from activated sludge in an urban wastewater treatment plant. The 16S rRNA gene sequence of W1 shared 99% similarity with several *Arthrobacter nicotianae* strains. It was deposited at the China General Microbiological Culture Collection Center under accession number CGMCC no. 4376. Strain W1 was regarded as a promising halotolerant microorganism which could survive and degrade phenol with salinity fluctuations in the range of 1% to 10% NaCl and in the presence of several salts (i.e., NaCl, KCl, Na_2_SO_4_, and K_2_SO_4_) (4). Furthermore, strain W1 could also degrade naphthalene and cometabolically degrade CA, DBF, and DBT with naphthalene as the primary substrate (5, 6). Compared with other reported *Arthrobacter* species, strain W1 has a wider substrate range and better environmental adaptability, especially in raw coking wastewater. The coking wastewater treatment efficiency was further improved using magnetically immobilized cells with activation zeolite, leading to much less toxicity than that of untreated wastewater. Thus, strain W1 might be used as an efficient microbial resource applied in the wastewater treatment process. The genome report of W1 may provide more comprehensive genetic information for the application of this bacterium in environmental pollution remediation in the future.

The genome of strain W1 was sequenced with an Illumina HiSeq-2000 platform (2- × 101-bp paired-end reads). The reads were assembled into 38 large contigs using Velvet 1.2.10 software, and the genome was functionally annotated through the Rapid Annotations using Subsystems Technology (RAST) annotation server (7, 8). There are 3,785,402 bp in total in the genome of strain W1. A total of 3,530 candidate protein-coding sequences (CDSs) were predicted, accounting for 86.6% of the total sequences. There were 72 RNA genes identified in the genome.

Seventy-four genes were annotated for the metabolism of aromatic compounds such as salicylate, gentisate, quinate, biphenyl, benzoate, and *p*-hydroxybenzoate in strain W1. In addition, several putative monooxygenases and dioxygenases were found in the genome, and there were 100 CDSs annotated for membrane transport, which would further explain its high degradation ability toward various aromatics. The genome information of strain W1 will provide further information for its practical application in wastewater treatment process.

### Nucleotide sequence accession numbers. 

This whole-genome shotgun project has been deposited at DDBJ/EMBL/GenBank under the accession number JWMD00000000. The version described in this paper is the first version, JWMD01000000.
